# Non-Coding RNAs as Potential Targets for Diagnosis and Treatment of Oral Lichen Planus: A Narrative Review

**DOI:** 10.3390/biom13111646

**Published:** 2023-11-13

**Authors:** Tae-Jun Kim, Yu Gyung Kim, Won Jung, Sungil Jang, Hyoung-Gon Ko, Chan Ho Park, Jin-Seok Byun, Do-Yeon Kim

**Affiliations:** 1 Department of Pharmacology, School of Dentistry, Kyungpook National University, Daegu 41940, Republic of Korea; 2 Department of Oral Medicine, Institute of Oral Bioscience, School of Dentistry, Jeonbuk National University, Jeonju 54896, Republic of Korea; 3 Department of Oral Biochemistry, Institute of Oral Bioscience, School of Dentistry, Jeonbuk National University, Jeonju 54896, Republic of Korea; 4 Department of Anatomy and Neurobiology, School of Dentistry, Kyungpook National University, Daegu 41940, Republic of Korea; 5 Department of Dental Biomaterials, School of Dentistry, Kyungpook National University, Daegu 41940, Republic of Korea; 6 Department of Oral Medicine, School of Dentistry, Kyungpook National University, Daegu 41940, Republic of Korea

**Keywords:** oral lichen planus, microRNA, long non-coding RNA, circular RNA, inflammation, T lymphocyte

## Abstract

Oral lichen planus (OLP) is a chronic inflammatory disease that is characterized by the infiltration of T cells into the oral mucosa, causing the apoptosis of basal keratinocytes. OLP is a multifactorial disease of unknown etiology and is not solely caused by the malfunction of a single key gene but rather by various intracellular and extracellular factors. Non-coding RNAs play a critical role in immunological homeostasis and inflammatory response and are found in all cell types and bodily fluids, and their expression is closely regulated to preserve normal physiologies. The dysregulation of non-coding RNAs may be highly implicated in the onset and progression of diverse inflammatory disorders, including OLP. This narrative review summarizes the role of non-coding RNAs in molecular and cellular changes in the oral epithelium during OLP pathogenesis.

## 1. Introduction

Oral lichen planus (OLP) is a chronic inflammatory disorder of the oral mucosa, commonly characterized by white striation-like lesions [1]. The global prevalence of OLP has been estimated as 1–2% [2], and OLP affects more women than men [3]. OLP lesions can be found bilaterally throughout the oral cavity and mainly involve the buccal mucosa, tongue, and gingiva [4]. In addition to the oral cavity, OLP can also present as a multisystemic disease with lesions in the genital and dermatological regions [5]. The age range of those affected varies, and the disease is uncommon in young people [3]. Additionally, OLP has been classified as an oral potentially malignant disease (OPMD) by the World Health Organization (WHO) [6].

Although the exact etiology of OLP remains largely unknown, accumulating evidence suggests that keratinocyte destruction mediated by hyper-activated T lymphocytes is a crucial step for OLP pathogenesis. Aberrant expression or unmasking of keratinocyte antigen may cause the initial inflammatory status of OLP. Subsequently, T lymphocytes (predominately CD8+ cells, but some CD4+ cells) invade the epithelium [7], and CD4+ T helper (Th) cells trigger the activation of CD8+ cytotoxic T lymphocytes (CTLs), causing the excessive production of pro-inflammatory cytokines and the apoptosis of basal keratinocytes [8].

Non-coding RNAs are RNA molecules that do not clearly demonstrate protein-coding activity. Non-coding RNAs have been simply divided into two groups depending on the transcript size, small non-coding RNAs (≤200 nt) and long non-coding RNAs (lncRNAs, ≥200 nt). Recently, it was suggested to classify non-coding RNAs into three groups [9]: (1) lncRNAs (>500 nt); (2) intermediate-sized non-coding RNAs (50–500 nt), including 5S rRNAs (~120 nt), tRNAs (76–90 nt), small nucleolar RNAs (snoRNAs, 60–300 nt), and small nuclear RNAs (snRNAs, ~150 nt); and (3) small non-coding RNAs (<50 nt), including microRNAs (miRNAs, 19–25 nt), small interfering RNAs (siRNAs, 20–29 nt), and piwi-interacting RNAs (piRNAs, 21–31 nt). Furthermore, recent advance in sequencing technology enabled the discovery of new classes of non-coding RNAs, such as circular RNAs (circRNAs) [10], tRNA-derived small RNAs (tsRNAs) [11], and novel small or long non-coding RNAs [12].

The molecular functions of some non-coding RNAs have been investigated in the context of OLP pathogenesis, particularly in keratinocytes and immune cells. In this review, we focus on several non-coding RNAs with established or prospective roles in OLP.

## 2. OLP Pathogenesis

### 2.1. Etiology and Initiation of OLP

Destruction of basal cells by T lymphocytes is generally accepted as a critical step for OLP development, but the etiology of OLP is still uncertain.

Although a pediatric OLP population has been reported [13], familial cases are very rare. OLP predominantly affects adults, especially women after the fourth decade of life [14]. However, some studies suggested that genetic predisposition may be involved in OLP onset. Wang et al. identified the chromosome 3p14-3q13 as a candidate region for OLP [15]. In addition, the aberrant expression of the major histocompatibility complex antigens HLA-DR was reported to possibly induce lymphocytic infiltration followed by interferon gamma (IFN-γ) production [16].

Although still debated, studies have suggested a possible association between cell-mediated host responses and exogenous viruses or microbial antigens in OLP development. Human papillomavirus (HPV), which can infect oral epithelial cells and cause oral cancer, has been linked to OLP pathogenesis. Some studies proposed that cell apoptosis in OLP resembles HPV infection. PCR analysis of oral tissue from OLP patients revealed significantly positive results for high-risk HPV types, such as HPV16 and HPV18 [17,18,19]. Bacteria have long been known to be deeply associated with T cell stimulation and immune responses. Considering that OLP is a disease mediated by T cells, research on oral microbial stimulation of T cells has been conducted. Studies indicate that OLP patients have demonstrated an imbalance in oral microbiota, which prompts immune responses [14]. For example, *E. coli*, a well-known gut microbe, is more abundant in OLP patients than in healthy controls and may activate the Toll-like receptor4 (TLR4)/nuclear factor-kappa B (NF-κB) signaling pathway to cause a Th17/Treg imbalance [20,21].

Psychosomatic factors should be seriously considered when exploring the etiology of OLP. Extremely high levels of stress, anxiety, and depression were detected in the OLP patients, compared with the negative control group [22], and stress has been regarded as the main factor contributing to sudden flare-ups in OLP patients [23]. In addition, frequent episodes of depression and anxiety as well as an increase in salivary cortisol in OLP patients are well-established indicators of the link between stress and OLP [24]. The relationship between OLP prevalence and individuals with psychological illnesses was also consolidated in a recent study [25].

OLP can be affected by other systemic diseases. Patients with chronic liver problems, including hepatitis C virus infection, are more likely to have ulcerative or erosive OLP [26]. In addition, collapsed endocrine function in diabetes may cause immunological dysregulation and may increase the risk of developing OLP [27]. Some anti-diabetic medications can cause patients with diabetes to develop an oral lichenoid lesion because of an allergic reaction [28]. Furthermore, Li et al. identified a comorbidity between OLP and thyroid gland diseases such as hypothyroidism and Hashimoto thyroiditis [29]. A relationship between OLP and dyslipidemia has also been suggested [30], and bowel conditions, including celiac disease, ulcerative colitis, and Crohn’s disease, have been occasionally linked to OLP [31].

Some non-coding RNAs are reported to be involved in the abnormal death of keratinocytes, which serves as the trigger for the initial response in OLP. For example, OLP patients have a downregulated level of miR-26a/b that protects OLP keratinocytes. The reduction of miR-26a/b induces Th1-related cytokines secretion and promotes apoptosis in oral keratinocytes, presumably contributing to the onset of OLP [32]. Although miR-27a/b levels were also downregulated in the oral epithelium and saliva of OLP patients, compared with healthy controls, decreased expression of miR-27 inhibited epithelial keratinocyte apoptosis [33,34,35]. Although the specific mechanisms through which miRNAs participate in the initial pathogenesis of OLP are not yet clear, recent research is exploring the impact of miRNAs on OLP, which will be discussed later in this review. Collectively, complex immunological mechanisms seem to be implicated in the etiopathogenesis of OLP.

### 2.2. Progression of OLP 

OLP predominantly affects non-keratinized tissues such as the buccal mucosa, tongue, and gums. As the lesions progress, the keratin layer of the affected oral mucosa undergoes abnormal formation that is characterized by the appearance of Wickham’s striae, a clinical hallmark of OLP [36]. It remains unclear exactly how these keratin layer modifications relate to the clinical manifestation of OLP lesions with Wickham’s striae. In OLP lesions, keratinocytes are activated to secrete pro-inflammatory cytokines such as TNF-α, IL-1β, IL-6, and IL-12 that contribute to the progression of OLP. Keratinocytes in OLP patients stimulate and differentiate nearby monocytes by releasing these cytokines [37,38].

The basement membrane (BM) of the oral epithelium is eventually disrupted as OLP lesions advance. This BM destruction is crucial in triggering a series of immune reactions, with T cells playing a significant role in perpetuating inflammation by more extensively infiltrating damaged tissues [39], and the compromising of the BM enables T cells to invade more forcefully [40]. Upon activation, CD4+ T cells can differentiate into subsets such as Th1, Th2, Th17, and Treg, each producing specific cytokines. The expression of IFN-γ, the characteristic cytokine of Th1, tends to increase in both the serum and lesion sites of OLP patients [41]. IFN-γ stimulates CD8+ T cells to destroy the epithelial cells of oral mucosa in OLP and inhibits the production of IL-4, the representative cytokine of Th2 [42]. The expression of IL-4 was also reported to be increased in OLP patients [41]. Nonetheless, the difference in IFN-γ between OLP and control groups was significantly more pronounced than the difference in IL-4, causing a Th1/Th2 cytokine imbalance. This Th1 cytokine bias can influence OLP susceptibility [41,43,44]. Th1-secreted IFN-γ and IL-2 may promote Th17 differentiation as OLP proceeds [43]. Th17 cell proportions were observed to be higher in OLP lesions (especially erosive type) [45], although their involvement in OLP pathophysiology requires more exploration. Foxp3-expressing Tregs also infiltrate OLP lesions and help the suppression of excessive immunological response in the oral mucosa [46,47,48].

Cytotoxic CD8+ T cells, which are mainly found near the BM in OLP lesions, are predominantly activated by cytokines released by CD4+ T cells, and by interacting with macrophages they induce keratinocyte apoptosis followed by BM destruction [49]. Although the self-antigen that triggers the initial response in OLP has not been clearly identified, BM keratinocytes have a potential to present antigens to CD8+ T cells via MHC I molecules, which can cause keratinocyte death [50]. In addition, keratinocyte apoptosis can be caused by the FAS-FASL interaction, which is initiated by TNF-α secreted from CD8+ T cells. The increased expression of Fas ligand and its receptor in OLP patients supports this hypothesis [51]. Cytotoxic substances such as perforin and granzyme that are mainly supplied by CD8+ T cells can also cause keratinocyte disruption, and a large number of perforin- and granzyme B-positive lymphoid cells have been observed in OLP lesions [52]. In line with this, NF-κB nuclear translocation in CD8+ T cells has been discovered around basal and suprabasal keratinocytes of OLP lesions, which is not seen in the normal epithelium [53]. Activation of the NF-κB pathway is known to increase perforin expression [54]. Furthermore, T cells in OLP lesions can produce and secrete RANTES, which induces mast cell degranulation and contributes to the chronicity of OLP [55]. More recently, the apoptosis of keratinocytes was demonstrated to be stimulated by T cell-derived exosomes [8].

**Figure 1:**
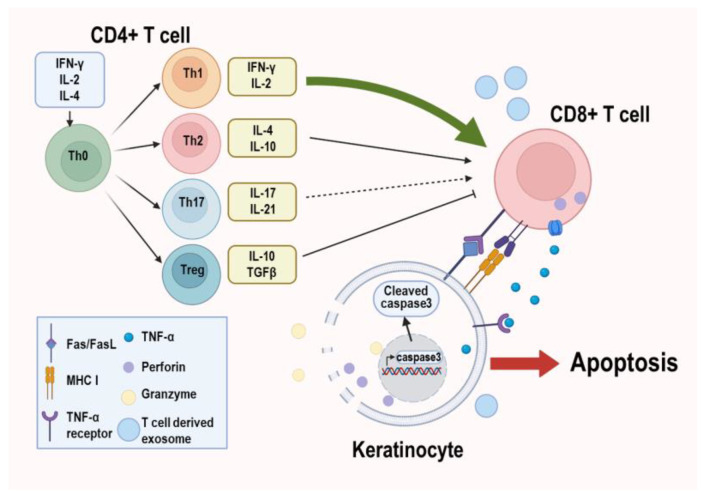
T cells–keratinocyte interaction in OLP. Upon stimulation, CD4+ T cells differentiate into Th1, Th2, Th17, and Treg subtypes. Th1-dominant imbalance of Th1/Th2 cytokine activates cytotoxic CD8+ T cells. Keratinocyte apoptosis can be stimulated in various ways, including antigen presentation, FAS-FASL interaction, T cell-derived cytotoxic substances, and exosomes.

Overall, as confirmed by single-cell RNA sequencing [56], various immune cells and cytokines/chemokines comprise the mucosal immune microenvironment of OLP and this immune ecosystem orchestrates the pathogenesis of OLP (Figure 1).

### 2.3. Molecular Features of OLP Pathogenesis

OLP is a multifactorial disease with a complex genetic basis, and although several genes have been implicated in the pathogenesis of OLP, no single key gene or antigen determinant that causes OLP has yet been found. Most of the current research focuses on genes involved in the control of the immunological response in T cells.

#### 2.3.1. Foxp3 (Forkhead Box Protein 3)

Tregs have been widely investigated in OLP because of their inhibitory roles in immune cell activation. The quantity of Tregs has been shown to be elevated in OLP lesions, and there is a negative correlation between Treg frequency and disease severity [48]. Foxp3 is a crucial transcription factor for the development and function of Tregs. Foxp3 is significantly more expressed in Tregs from OLP patients’ lesions than in healthy controls [57]. Mechanistically, Foxp3 inhibits NF-κB signaling by promoting the expression of miR-146a. Furthermore, Foxp3 seems to inhibit the activity of other CD4+ T cells, including Th1 and Th17 cells, by lowering IFN-γ and IL-17 levels [47]. Genetic variations in Foxp3, which affects the activity of Tregs and immunological tolerance, have also been studied in relation to OLP.

#### 2.3.2. mTOR (Mammalian Target of Rapamycin)

mTOR plays a central role in T cell metabolism and immunological responses. As a sensor of the metabolic environment, mTOR upregulates glycolytic flux by inducing LDHA phosphorylation and increasing the expression of Glut1 and PDH. Treatment with rapamycin, a specific inhibitor of mTOR, showed that mTOR restrained both T cell proliferation and DNA replication, stimulated apoptosis, and affected Th1/Th2 and Th17/Treg ratios, suggesting that mTOR is a critical modulator in T cell fate decision [58]. mTOR and the transcription factor 4E-BP1 are reported to be substantially phosphorylated in the T cells of OLP patients. Dysregulated mTOR signaling in T cells decreased the apoptosis rate, presumably leading to substantial degradation of keratinocyte [59].

#### 2.3.3. HIF-1α (Hypoxia-Inducible Factor-1 Alpha)

HIF-1α plays a significant role in immune functions by controlling T cell development and proliferation. Interestingly, patients with OLP showed abnormal expression of HIF-1α and its regulator, phospholipase D2 (PLD2), and this promotes T cell proliferation and phenotypic differentiation into the pro-inflammatory state via glycolysis upregulation, independently of the mTOR pathway [60]. In addition, the expression of RTP801 and VEGF, which are other target genes of HIF-1α, were significantly decreased, which presumably contributes to apoptosis and inflammatory response in the oral mucosa [61]. Furthermore, HIF-1α gene polymorphisms have been reported to be associated with risk of OLP [62].

#### 2.3.4. TRIM21 (Tripartite Motif-Containing Protein 21)

TRIM21 is a well-studied autoantigen whose expression is induced by IFN-γ and is a key gene in autoimmune disorders. Notably, T cells from OLP patients possess higher levels of TRIM21 than those of healthy people [63]. Elevated levels of TRIM21 in T cells (mainly in CD3+ T lymphocytes) cause enhanced proliferation and infiltration in OLP lesions. Mechanistically, the overexpression of TRIM21 caused abnormal expression of pro-inflammatory cytokines and chemokines via the TRIB2-MAPK signal axis and the NF-κB signaling pathway, eventually leading to the Th1/Th2 imbalance [64,65]. Interestingly, TRIM21 is overexpressed in COVID-19 patients, which may make them more susceptible to developing OLP, according to a recent study [66].

## 3. Implications of miRNA for OLP Pathogenesis

### 3.1. Molecular Functions of miRNA

Among small non-coding RNAs, miRNA has been the subject of the most research. miRNAs are small, single-stranded transcripts that function as key regulators in diverse pathophysiologies. Most research to date has demonstrated that miRNAs interact with a specific sequence on their target mRNAs to cause translational repression as well as mRNA degradation. In animal cells, the miRNA-guided target RNA recognition occurs via the 5′ seed region (6–8 nucleotides at the 5′ end of the miRNA) [67]. miRNAs are known to predominantly interact with the 3′ untranslated region (UTR) of target mRNAs for post-transcriptional gene regulation but can also bind to the 5′ UTR or coding sequence of target mRNAs. Although miRNAs primarily downregulate gene expression, several miRNAs occasionally induce translation upregulation of target mRNAs. For example, let-7 activates translation of target mRNAs during cell cycle arrest but represses translation in proliferating cells [68]. In addition, a KLF4-miR-206 feedback loop influences protein synthesis in both healthy and cancer cells in opposing ways [69]. Furthermore, miR-10a promotes the translation of ribosomal protein mRNAs by binding to them to possibly improve global protein synthesis [70].

Interestingly, miRNA-mediated transcriptional controls have also been reported. miRNA can interact with the promoter region of the protein-coding genes to mediate transcriptional regulation [71]. For example, miR-552 suppresses *CYP2E1* transcription by forming hybrids with a cruciform structure within the *CYP2E1* promoter [72]. In addition, miR-24-1 increases overall gene transcription by binding and activating its target enhancers [73]. Chromatin status at target promoters can also be switched into a silent state by let-7 during cellular senescence [74].

Accumulating evidence suggests that miRNAs have extracellular functions. In specific pathophysiological circumstances, specific miRNAs can be released from cells [75]. These extracellular miRNAs are found in various bodily fluids and may act as disease indicators [76]. Furthermore, a recent study showed that miR-711 binds to the extracellular S5–S6 loop of TRPA1, working as an itch mediator and ion channel modulator [77]. Collectively, research on miRNA is rapidly growing, although this remains an extremely complex field in terms of gaining a comprehensive understanding.

### 3.2. Roles and Biological Relevance of miRNAs in OLP

miRNAs may now be recognized as novel participants in immunological homeostasis and inflammatory illnesses such as OLP. In a microarray investigation of the miRNAs from OLP patients and healthy controls, the expression of almost 70 different types of miRNAs was shown to dramatically change. With further investigation, the expression of at least nine miRNAs was experimentally proven to be elevated or depressed in the blood, saliva, or lesions of OLP patients, compared with that in healthy individuals [78]. Of these nine miRNAs, we focused on three miRNAs that are particularly important in the immune system and OLP pathogenesis.

#### 3.2.1. hsa-miR-155

miR-155 is transcribed from the host gene *MIRHG155*, which is also known as the B cell integration cluster, located on chromosome 21 [79]. Pre-miR-155, a 65 nt long stem-loop precursor, is converted from pri-miR-155 in the nucleus and then further processed in the cytoplasm to create the miR-155 duplex, a 22-nucleotide structure with -5p and -3p strands. The level of miR-155 is dynamically modulated by several upstream factors, including AP-1 [80], HIF-1α [81], protein kinase C (PKC), and NF-κB [82].

miR-155 is highly expressed in immune cells, such as T lymphocytes, B lymphocytes, NK cells, macrophages, and dendritic cells, and plays a critical role in immune responses ranging from inflammation to immunological memory [83]. For example, miR-155 shows a strongly enhanced expression upon T cell activation, which contributes to CD8+ T cell proliferation and survival, whereas miR-155 deficiency impairs antiviral and antitumor response in CD8+ T cells [84,85]. Mechanistically, transcriptome analysis revealed that type I interferon signaling and sensitivity to the interferon antiproliferative effect were increased by miR-155 depletion [86]. In addition, miR-155 downregulates the expression of multiple negative elements of Akt and Stat5 signaling, including the inositol 5-phosphatase Ship1, protein tyrosine phosphatase Ptpn2, and suppressor of cytokine signaling 1 (Socs1), to augment T cell activation and cytokine response [87]. miR-155 expression is highly upregulated upon activation in B cells, and miR-155 loss disrupts the germinal center response by downregulation of cytokine (TNF and LT-α) secretion, reduced immunoglobulin G1 (IgG1) production, and inefficient affinity maturation [88]. The transcription factor PU.1 and activation-induced deaminase are representative miR-155 targets in B cells, and miR-155-mediated post-transcriptional regulation of these genes is required for optimal B cell proliferation, development, and survival [89,90]. miR-155 is also important for the innate immune response. In monocytes/macrophages, miR-155 expression is induced by microbial constituents and pro-inflammatory mediators through c-Jun N-terminal kinase (JNK) activation [91,92], and miR-155-mediated translational silencing of negative regulators involved in lipopolysaccharide (LPS)/NF-κB signaling, including IκB kinase ε (IKKε), the Fas-associated death domain protein, and the receptor (TNFR superfamily)-interacting serine-threonine kinase 1 (Ripk1), stimulates cytokine production in macrophages [93].

The significance of miR-155 in the immune system led to extensive studies of the role of miR-155 in diverse inflammatory diseases and autoimmune disorders. miR-155 shows increased expression in the synovial compartment of patients with rheumatoid arthritis (RA), a chronic autoimmune disease. Mice lacking miR-155 are resistant to synovial inflammation and cartilage/bone destruction, indicating that miR-155 is crucial for RA pathogenesis [94]. miR-155 expression in Sjögren’s syndrome (SS), a chronic autoimmune disease that demonstrates dysfunction in moisture-producing glands, is controversial. While Shi et al. reported the downregulation of miR-155 in peripheral blood mononuclear cells (PBMCs) of SS patients [95], Chen et al. observed the overexpression of miR-155 in exactly the same condition [96]. Notably, miR-155 seems to be strongly upregulated in SS patients with decreased salivary flow compared with those with normal salivary flow [97]. miR-155 silencing suppressed the apoptosis and inflammation of salivary gland epithelial cells, suggesting the relevance of miR-155 in the pathogenesis of SS [98].

The expression of miR-155 was positively correlated with the severity of OLP. During OLP progression, miR-155 and IFN-γ reinforce each other, which encourages Th1 cell polarization and upsets the Th1/Th2 equilibrium [99]. In addition, miR-155 directly targeted endothelial nitric oxide synthase (eNOS) in PBMCs from subjects with OLP, causing the inhibition of NO production [100]. In OLP-associated fibroblasts, miR-155 augmented the release of IL-6 and IL-8 and reduced SOCS1 expression [101]. Collectively, a cascade of miR-155-mediated responses within various cell types is implicated in the initiation and progression of OLP.

#### 3.2.2. hsa-miR-21

In addition to miR-155, miR-21 was also reported to be increased in OLP patients [102]. miR-21 was one of the first miRNAs to be recognized as being produced by RNA polymerase II [103]. Although several upstream factors, including AP-1 [104], STAT3 [105], and C/EBPβ [106], have been identified as inducing miR-21 expression, the transcriptional regulation of miR-21 has yet to be fully characterized [107].

In immune cells, miR-21 expression is significantly elevated during maturation or activation from progenitors such as monocyte-derived dendritic cell differentiation [108] and LPS-mediated macrophage activation [109]. miR-21 deficiency promoted M1 polarization in macrophages through the induction of IFN-γ-STAT1 signaling [110]. Also, macrophages lacking miR-21 enhanced the expression of the miR-21 target gene, MKK3, inducing the upregulation of p38-CHOP and JNK signaling and causing ER-stress-mediated apoptosis [111]. An antiapoptotic function of miR-21 was also observed in T cells. miR-21 was significantly increased by T cell receptor engagement of naïve T cells [112]. Consequently, memory T cells express more miR-21 than naïve T cells do, and miR-21 is more abundant in activated CD4+ and CD8+ T lymphocytes in comparison with their dormant counterparts [113]. As a homeostatic balancing mechanism in the immune system, immune cell activation is tightly linked to programmed cell death. miR-21 inhibits activation-induced apoptosis in T cells through directly targeting Tipe2 [114]. In addition, miR-21 was reported to function as a key factor in determining the precise balance between Th1 and Th2 responses. miR-21-deficient mice showed increased expression of the Th1 cytokine IFN-γ and decreased expression of Th2 cytokine IL-4 to cause Th1 polarization in vivo [115].

Similar to miR-155, miR-21 plays critical roles in many inflammatory pathways, and miR-21 dysregulation is linked to diverse disorders. Upon LPS stimulation in human PBMCs, induced miR-21 negatively controls the inflammatory response by suppressing the expression of PDCD4, which then activates NF-κB and downregulates IL-10 [116]. In contrast, miR-21 has been identified to be increased in epidermal lesions of psoriasis patients to promote T cell-derived psoriatic skin inflammation through the activation of the IL-6-STAT3 pathway [117,118], indicating that miR-21 can exert either pro-inflammatory or anti-inflammatory effects, depending on circumstances. Collectively, miR-21 plays pivotal roles in various pathologic conditions.

Given that altered levels of miR-21 in OLP was already reported, the amount of miR-21 in saliva has gained increasing attention as a diagnostic and prognostic indicator [102,119]. However, the cell-type-specific expression and function of miR-21 during OLP progression has not been extensively studied so far. The specific molecular mechanism of miR-21 and its significance in the pathophysiology of OLP need to be examined in the future.

#### 3.2.3. hsa-miR-146a

Initially discovered in 2002 in mouse heart tissue, miR-146 was proven to have a human counterpart by a further study [91,120]. The two miRNAs miR-146a and miR-146b, which are respectively found on human chromosomes 5 (5q33.3) and 10 (10q24.32), constitute the miR-146 family. Only two nucleotides separate miR-146a and miR-146b at the 3′ part of the mature sequence, rather than the seed region, indicating that they presumably share target transcripts. While both miR-146a and miR-146b were commonly shown to be induced by the NF-κB signaling pathway [91], their responses to cytokine stimulation differ, suggesting that they are differentially controlled [121]. Although research on the miR-146 family is expanding, published material on miR-146a surpasses that on miR-146b by approximately five-fold [122].

miR-146a controls the innate immune system to balance the inflammatory response. In the THP-1 human monocyte cell line, TLR stimulation by LPS triggers NF-κB overexpression to induce the expression of miR-146a, which subsequently regulates the expression of tumor necrosis factor receptor-associated factor 6 (TRAF6) and interleukin-1 receptor-associated kinase 1 (IRAK1) to provide negative feedback for TLR signaling [91]. The negative regulation of miR-146a to IRAK1 was also confirmed in PBMCs of psoriasis patients [123] as well as in IL-17-producing T cells of RA patients [124]. The inhibitory relationship between miR-146a and TRAF6 was additionally examined in macrophages of polymyositis/dermatomyositis [125]. In the pathogenesis of systemic lupus erythematosus, miR-146a serves as a negative regulator of the type-I interferon pathway [126].

**Figure 2:**
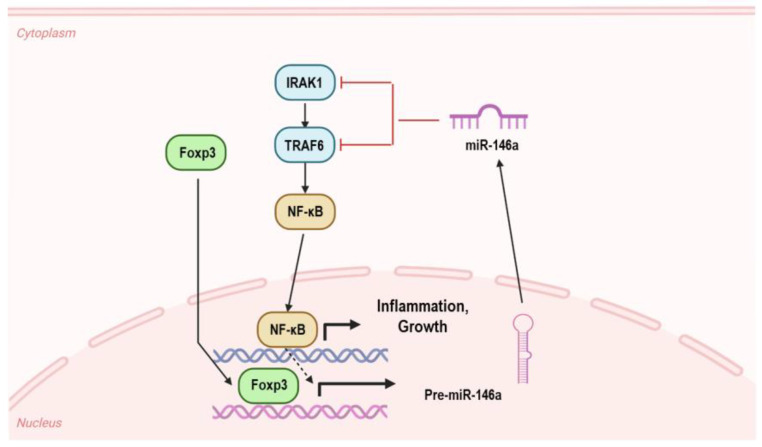
Underlying molecular mechanisms of miR-146a in the microenvironment of OLP. Foxp3 can induce the expression of miR-146a in both Tregs and keratinocytes. miR-146a mediates inflammatory response, proliferation, and apoptosis by targeting IRAK1 and TRAF6.

Unfortunately, few clues are currently available concerning the roles of miR-146a in OLP. A higher expression of miR-146a was found in local lesions in the OLP group than that in a healthy control group. However, the miR-146a expression in peripheral blood CD4+ T cells did not substantially differ between the OLP patients and the control group, indicating that miR-146a may have a greater role in the local immunological responses of OLP [127]. By blocking NF-κB signaling, Foxp3-induced expression of miR-146a in Tregs enhanced their suppressive capabilities [47]. Foxp3-mediated miR-146a induction was also reported in keratinocytes. miR-146a can mediate both cell proliferation and apoptosis through targeting TRAF6 [128]. Collectively, these findings indicate that miR-146a may play critical roles in the microenvironment of OLP (Figure 2).

## 4. Implications of lncRNA for OLP Pathogenesis

### 4.1. Molecular Functions of lncRNA

Similar to mRNAs, many lncRNAs are transcribed by RNA polymerase II and possess 5′ caps and 3′ poly A tails. However, while mature mRNAs are mainly present in the cytosol to be translated, lncRNAs are more frequently found in the nucleus [129]. lncRNA genes appear to be less evolutionarily conserved, have fewer and longer exons, and are expressed at lower levels than mRNAs [130]. In addition, lncRNAs undergo splicing far less frequently than mRNAs [131]. A significant portion of lncRNAs are exported to the cytoplasm via similar export mechanisms as mRNAs. lncRNAs are then likely sorted, either by being disseminated throughout the cytoplasm and interacting with various RNA-binding proteins, or by being assigned to particular organelles [132]. Approximately 70% of cytoplasmic lncRNAs are bound to ribosomes, but their precise function has yet to be understood [133].

Like miRNAs, lncRNAs can modulate transcription, splicing, mRNA stability, and translation of target genes. Several lncRNAs mediate chromatin regulation by interacting with chromatin modifiers. For example, lncRNA HOTTIP directly binds to WD repeat-containing protein 5 (WDR5) to recruit WDR5/MLL complexes to the promoter region of the HOXA gene, inducing histone H3 Lys4 trimethylation (H3K4me3) and gene transcription [134]. lncRNAs have been proposed to directly interact with DNA to form hybrid structures called triplexes (triple helices) or R-loops that modulate chromatin accessibility [135]. lncRNAs are also involved in post-transcriptional, translational, and post-translational steps for gene regulation. For example, lncRNA PNCTR sequesters a significant fraction of PTBP1 in the perinucleolar compartment, suppressing PTBP1-mediated mRNA splicing and eventually boosting cell survival [136]. lncRNA AF087999 (1/2-sbsRNAs) binds to and transactivates Staufen 1 (STAU1), which associates with double-stranded RNA for degradation, thereby reducing the abundance of STAU1-mediated mRNA decay targets [137]. lncRNA PNUTS operates as a competitive sponge for miR-205, enabling the upregulation of ZEBs during the epithelial–mesenchymal transition [138].

Surprisingly, recent evidence demonstrated that a group of lncRNAs can be translated into polypeptides. Functional peptides expressed by lncRNAs have been a useful study topic because of their potential significance in fundamental pathophysiologies [139]. Moreover, lncRNA-derived peptides that are loaded on surface-bound MHC proteins in tumor cells are promising targets for cancer immunotherapy [140].

### 4.2. Roles and Biological Relevance of lncRNAs in OLP

Unlike miRNAs, lncRNAs have been rarely studied in OLP. Transcriptome profiling was used to accomplish genome-wide lncRNA annotation in psoriasis [141], a chronic inflammatory condition with a similar immunopathogenic (T cell-mediated) mechanism to OLP, but lncRNA functional research in OLP is far more limited.

#### 4.2.1. SPRR2C (Small Proline-Rich Protein 2C)

The human SPRR genes are a multigene family consisting of proteins and non-coding RNAs, located within the epidermal differentiation complex on chromosome 1q21 [142]. Protein members of the SPRR family function as precursors of the cornified cell envelope, a component in the outermost layers of stratified squamous epithelia, which is responsible for the protective barrier of the skin [143]. In addition, SPRR genes have been regarded as keratinocyte differentiation markers because of their induced expression during the normal differentiation process of epidermal keratinocytes [144,145]. Notably, the SPRR family includes the SPRR2C lncRNA that has a T at position 141, whereas other protein coding SPRR2 members (SPRR2A, SPRR2B, SPRR2D, SPRR2E, SPRR2F, and SPRR2G) possess a C at this position. This single nucleotide change leads to the conversion from the codon (CAG) encoding glutamine into a stop codon (TAG), generating a pseudogene SPRR2C.

SPRR2C has been suggested as a biomarker for anemia after kidney transplantation [146]. However, the relevance of SPRR2C has been more strongly supported in skin pathophysiologies. As a skin-expressed lncRNA, SPRR2C is prominently induced in both psoriasis and atopic dermatitis lesions. Interestingly, the expression of SPRR2C is considerably increased in the non-lesional skin of both psoriasis and atopic dermatitis but to a lesser extent than in lesional skin [147]. Consistently, in the psoriatic cell models, keratinocytes treated with IL-22 or M5 (IL-17A, TNF-α, IL-1, IL-22, and oncostatin-M) also showed increased expression of SPRR2C [148,149]. Importantly, two independent studies both demonstrated that SPRR2C silencing suppressed cell proliferation, increased cell death, and inhibited the pro-inflammatory cytokine production presumably by influencing the PI3K/AKT/mTOR pathway. In addition, the level of endogenous SPRR2C transcript dynamically changes with age and SPRR2C appears to control keratinocyte differentiation [150]. These data collectively suggest that SPRR2C can be a critical hub gene in homeostatic regulation of keratinocytes and that SPRR2C may have potential roles in OLP progression.

#### 4.2.2. FABP5P3 (Fatty Acid Binding Protein 5 Pseudogene 3)

Fatty-acid-binding proteins (FABPs) are small, structurally conserved cytosolic proteins that chaperone hydrophobic ligands and transport them throughout cellular compartments [151]. Ten FABP isoforms are expressed in humans and most are ubiquitously expressed throughout tissues [152]. Among these, FABP5, also known as epidermal FABP, keratinocyte-type FABP, or psoriasis-associated FABP, is widely detected in the skin, tongue, adipocyte, mammary gland, brain, stomach, intestine, kidney, liver, lung, heart, skeletal muscle, testis, retina, lens, spleen, and placenta.

**Figure 3:**
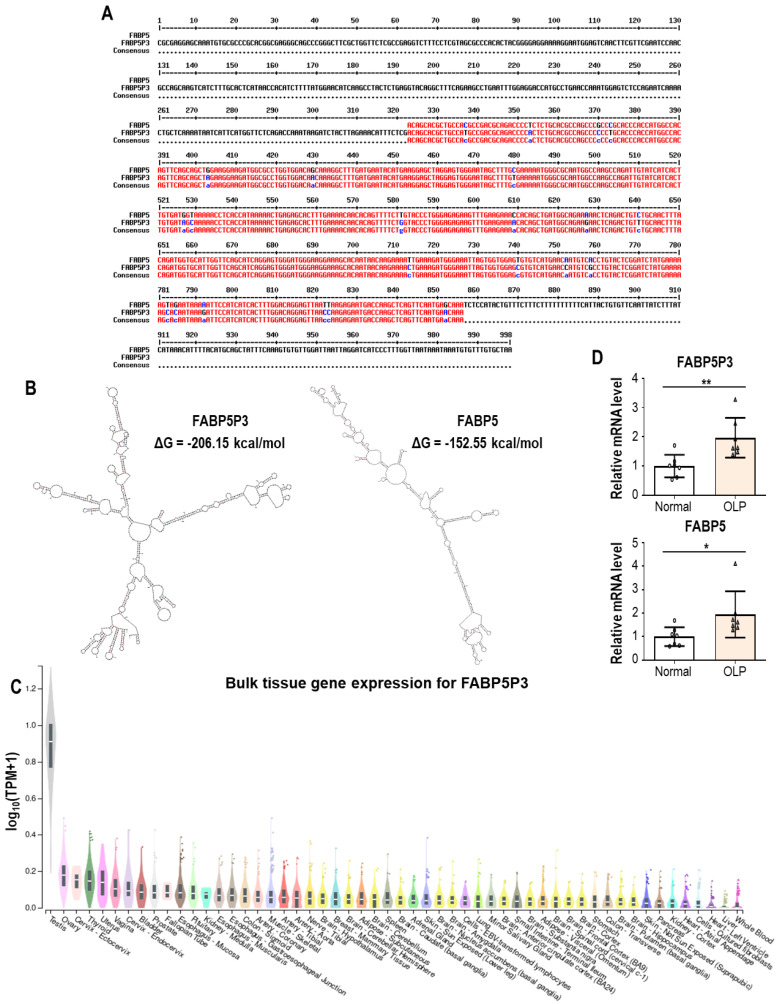
Sequence and expression analysis of FABP5P3. (**A**) Sequence comparison between FABP5P3 and FABP5, generated using Multalin [155]. (**B**) Predicted RNA secondary structures for the lncRNA FABP5P3 and FABP5 mRNA, based on free energy calculations via mfold program. (**C**) Expression of FABP5P3 across tissues, taken from the GTEx Portal. TPM, transcripts per million. (**D**) FABP5P3 and FABP5 expression in normal oral and OLP epithelium. Data were extracted and analyzed from the NCBI GEO profile database (GEO accession: GSE52130). Expression was normalized with that of ACTB. Statistical significance was determined by unpaired *t*-test. * *p* < 0.05 and ** *p* < 0.01.

FABP5P3 is similar in sequence to FABP5 mRNA (Figure 3A) but FABP5P3 (856 nt) is reportedly longer than FABP5 mRNA (676 nt). According to RNA secondary structure prediction analysis [153], FABP5P3 RNA appears to fold in a different way compared with FABP5 mRNA (Figure 3B). When we analyze the distribution of FABP5P3 expression across tissues via the GTEx Portal (https://www.gtexportal.org/, accessed on 8 September 2023), FABP5P3 is most likely strongly expressed in the testis (Figure 3C). However, FABP5P3 has been the subject of functional research in tissues other than the testis. In kidney, FABP5P3 could improve TGFβ1-stimulated deregulation of fatty acid oxidation (FAO) and renal fibrosis. Mechanistically, FABP5P3 may engage in competitive miR-22 binding with the mRNAs of CPT1A, NCOA1, and RXRA, three crucial genes implicated in FAO, to offset the inhibitory effects of miR-22 on FAO. Consequently, FABP5P3 reversed TGFβ1-induced FAO suppression in proximal tubular epithelial cells and ultimately alleviated renal fibrosis [154].

Two independent studies both demonstrated that FABP5P3 supports cell proliferation. According to Zhu et al., the expression of FABP5P3 is upregulated in hepatocellular carcinoma (HCC), and FABP5P3 expression levels are inversely correlated with survival rates [156]. FABP5P3 promoted HCC progression by counteracting miR-589-5p that has been reported as a tumor suppressor. Notably, FABP5P3 was recently shown to facilitate keratinocyte proliferation and skin inflammation [157]. By recruiting human antigen R (huR), FABP5P3 inhibited KMT2C mRNA degradation. In psoriatic lesions, lncRNA FABP5P3 was found to be upregulated, causing the accumulation of KMT2C mRNA and protein. In turn, abundant KMT2C protein activates the transcription of PIK3R3, the critical gene for cell proliferation and downstream inflammatory response. Although OLP is a T cell-mediated chronic inflammatory disease with excessive destruction of the basal keratinocytes, epidermal hyperplasia is also frequently observed. The increased proliferation of keratinocytes has been regarded as a compensatory mechanism so far. Given that lncRNA FABP5P3 seems to be upregulated in OLP lesions (Figure 3D), based on analyzing the gene expression profile (GSE52130) [158], increased FABP5P3 expression could contribute to keratinocyte hyperproliferation in OLP, which must be confirmed in the future.

#### 4.2.3. PRINS (Psoriasis Susceptibility-Related RNA Gene Induced by Stress)

The name of lncRNA PRINS reflects the change in its expression by external stimuli or disease status. PRINS showed higher expression in non-lesional epidermis of psoriasis, compared with that in both psoriatic lesions and healthy skin, suggesting that PRINS may be involved in psoriasis susceptibility. In addition, PRINS is upregulated upon exposure to stress such as ultraviolet irradiation, viral infection, and translation blockade [159].

PRINS demonstrates variable expression patterns in different tissues. While PRINS is strongly detected in the gut, lungs, lymph nodes, uterus, testicles, and skin, it is present at low levels in the breast, kidney, stomach, and gallbladder. PRINS is almost absent in the brain. In skin, a higher level of PRINS is present in the epidermis than in the dermis. PRINS is abundant in nucleolar and perinuclear areas, but cytoplasm also homogeneously contains PRINS to a lesser level [160].

Accumulating evidence suggests that PRINS has a key role in keratinocyte pathophysiologies. Szegedi et al. found that G1P3, a downstream target gene of PRINS, was dramatically upregulated in hyperproliferative lesional psoriatic epidermis compared with that of healthy controls. Considering the antiapoptotic activity of G1P3, the PRINS–G1P3 axis seems to suppress spontaneous keratinocyte apoptosis and contribute to the development of psoriasis [161]. In addition, it was previously reported that the level and the intracellular localization of nucleophosmin (NPM), one of the identified binding partners of PRINS, were altered in lesional psoriatic epidermis [160]. Given that NPM modulates diverse cellular processes, deregulation of the PRINS–NPM axis would be involved in psoriasis pathogenesis. Interestingly, PRINS knockdown did not significantly influence cell survival when keratinocytes were maintained in favorable conditions. However, under serum starvation, the keratinocyte survival rate was clearly decreased by PRINS silencing, suggesting that PRINS plays key roles in cellular stress response [162].

**Figure 4:**
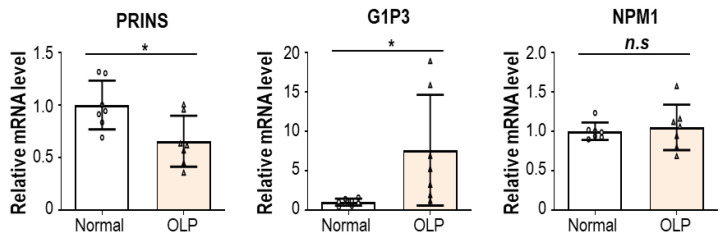
PRINS, G1P3, and NPM1 expression in normal oral and OLP epithelium. Expression of PRINS, G1P3, and NPM1 was analyzed. Data were extracted and analyzed from the NCBI GEO profile database (GEO accession: GSE52130). Expression was normalized with that of ACTB. Statistical significance was determined by unpaired *t*-test. * *p* < 0.05. n.s., not significant.

Expressional analysis or functional study of PRINS in OLP has yet to be performed. In contrast to psoriasis, lncRNA PRINS expression seems to be downregulated in OLP lesions (Figure 4), suggesting that PRINS has different roles in OLP progression. Indeed, while the expression patterns of PRINS and its target genes, G1P3 and NPM, were positively correlated in psoriasis patients [163], their expression appears to be uncoupled in OLP lesions (Figure 4). Given that PRINS demonstrates an anti-inflammatory function via the destabilizing mRNAs of IL-6 and CCL5 [164], it would be necessary to confirm whether the decreased expression of PRINS results in the overproduction of pro-inflammatory cytokines in OLP pathogenesis.

#### 4.2.4. DQ786243

A newly discovered lncRNA called DQ786243 was initially found to be upregulated in liver cancer [165]. Although functional analysis of DQ786243 has not been precisely conducted in inflammatory conditions, Qiao et al. reported that patients with clinically active Crohn’s disease exhibited substantial DQ786243 overexpression [166]. Thus, DQ786243 may be connected to Crohn’s disease severity and appears to positively regulate CREB and Foxp3 expression, which control Treg function.

Autoimmune and inflammatory diseases depend on Treg functionality. In patients with multiple sclerosis, the suppressive activity of Tregs is significantly reduced [167]. Similarly, RA patients showed a substantial decrease in the activity of Tregs to suppress T cell responses [168]. Furthermore, in OLP patients, Tregs were functionally compromised [169]. Consistent with a study by Qiao et al. [166], Wang et al. demonstrated that DQ786243 controls the activation and operation of CD4+ Tregs by positively influencing Foxp3 expression [47]. The authors found that the expression of both DQ786243 and Foxp3 was upregulated in CD4+ cells from OLP patients, and dysregulation of DQ786243 and Foxp3 expression might be linked to the development of OLP. Furthermore, the DQ786243–Foxp3 axis seems to induce miR-146a that is involved in OLP pathogenesis.

## 5. Implications of circRNA for OLP Pathogenesis

### 5.1. Molecular Functions of circRNA

circRNA is a novel form of single-stranded non-coding RNA with a covalently closed loop instead of free 3′ and 5′ ends. This circular structure is generated by a specialized process called backsplicing [170]. Unlike linear RNA, circRNAs are more protected from the degradation by exonucleases and consequently have an extended half-life [171]. Recently, this structural advantage of circRNA has been used for translation platforms or RNA vaccines [172,173,174]. Since the first discovery of endogenous mammalian circRNA in 1991 [175], many circRNAs have been detected in various cell types and they are mainly located in the cytoplasm inside cells [10].

circRNAs have a wide range of regulatory functions and can serve as a miRNA sponge. For example, circRNA CDR1as binds to miR-7, the critical miRNA for neuronal differentiation, and overexpression of CDR1as impairs brain development [10]. In addition, circRNA Sry and ciRS-7 act as antagonists of miR-138 and miR-7, respectively [176]. More recently, circRNA circBCAR3 was shown to support proliferation, migration/invasion, and ferroptosis of esophageal cancer cells through sponging miR-27a [177]. circRNA can be involved in gene expression regulation by functioning as a protein sponge. The transcript of the splicing factor MBL can be circularized, and this circRNA (circMbl) interacts with its protein product MBL to disrupt pre-mRNA splicing [178]. circRNAs can act as a scaffold during apoptosis. By binding to FoxO3 protein, circRNA circ-Foxo3 prevents FoxO3 degradation. Accumulated FoxO3 enhanced expression of its downstream target Puma, which results in apoptosis [179]. circRNAs are also involved in post-transcriptional regulations such as mRNA stability and translation control. For instance, circXPO1 protected CTNNB1 mRNA from degradation by interacting with IGF2BP1, thereby contributing to lung adenocarcinoma progression [180].

With the advance of high-throughput sequencing technology, more circRNAs have been discovered. Although circRNAs were once considered to be byproducts of abnormal RNA splicing, diverse critical roles of circRNAs have been recently demonstrated. The expression, molecular function, and regulatory mechanism of circRNAs have been explored, and circRNA research is now growing exponentially.

### 5.2. Roles and Biological Relevance of circRNAs in OLP

A recent study by Song et al. analyzed the expression profile of circular RNAs in OLP [181]. The authors performed high-throughput circRNA sequencing and discovered 135 circRNAs that had significantly altered expression levels in the oral mucosa of OLP patients compared with those in healthy controls (83 upregulated, 52 downregulated). The GO enrichment analysis revealed that circRNAs with upregulated expression are mostly involved in biological functions that include T cell-mediated cytotoxicity, extracellular stimulation response, and antigen processing and presentation. According to the KEGG pathway analysis, natural killer cell-mediated cytotoxicity, B-cell receptor signaling pathway, cGMP-PKG signaling pathway, and T cell receptor signaling pathway seemed to be dysregulated in OLP. To better understand the functions of circRNAs in OLP pathogenesis, further studies need to be performed. It is important to determine the type of cell that expresses circRNAs differently. In addition, it is necessary to undertake functional research on each circRNA.

## 6. Conclusions

OLP is a persistent oral mucosal inflammatory condition that can cause discomfort, burning, swelling, and bleeding. OLP impairs the development of social interactions, increases the risk of depression, and significantly lowers the quality of life. Considering that OLP has been defined as a premalignant disorder, early detection and appropriate treatment of OLP are imperative. OLP is extremely complex, involving many different types of cells, genes, non-coding RNAs, and extracellular factors in its development and progression, making it challenging to treat. In particular, the role of non-coding RNAs, especially lncRNAs, in OLP pathogenesis is largely unexplored. A fundamental understanding of OLP requires a detailed examination of the expression and regulation mechanisms of various genes in pathological situations, and a closer look at the non-coding RNAs that regulate these genes. Therefore, further investigation into the role of key non-coding RNAs in OLP lesions will provide novel insights into OLP pathophysiologies and help find a potential new strategy against OLP. As the role of non-coding RNAs in OLP continues to be explored, notably in immunological responses and inflammation, the expression or function of non-coding RNAs may be the subject of early diagnosis or novel treatment approaches. Non-coding RNAs hold great promise as biomarkers or therapeutic targets in OLP.

## Data Availability

Not applicable.
